# Pyrococcus furiosus Argonaute coupled PCR assay for accurate discrimination between the MS-H vaccine strain and clinical isolates of Mycoplasma synoviae

**DOI:** 10.1371/journal.pone.0351464

**Published:** 2026-07-28

**Authors:** Yanli Zhao, Yinling Wang, Ge Song, Houqiang Luo, Liyan Dong, Qingsong Han, Mengling Yang, Jing Pan, Hongxia Jiang

**Affiliations:** 1 College of Animal Science, Wenzhou Vocational College of Science and Technology, Wenzhou, China; 2 Guangdong Key Laboratory for Veterinary Pharmaceutics Development and Safety Evaluation, College of Veterinary Medicine, South China Agricultural University, Guangzhou, China; 3 Guangdong Laboratory for Lingnan Modern Agriculture, Guangzhou, China, South China Agricultural University, Guangzhou, China; Hirosaki University Graduate School of Medicine, JAPAN

## Abstract

*Mycoplasma synoviae* (MS) is a significant avian pathogen responsible for arthritis, tenosynovitis, airsacculitis, and abnormal eggshell apex syndrome in chickens, posing a substantial threat to the poultry industry. While the attenuated MS-H vaccine has proven effective and is widely implemented in poultry flocks. However, distinguishing the MS-H vaccine strain from wild-type strains remains a persistent challenge. Recently, Pyrococcus furiosus Argonaute (PfAgo) nucleases have garnered considerable attention due to their capacity for single-nucleotide discrimination. Leveraging the A367G SNP within the MS *obg* gene, we developed a novel identification method that integrates PfAgo-mediated cleavage with PCR amplification. Through systematic optimization of PfAgo cleavage substrates and PCR primers, this approach achieved detection sensitivities of 1 × 10^3^ copies/µL for the MS-H vaccine strain and 1 × 10^4^ copies/µL for wild-type strains. Validation using 12 clinical samples resulted in the accurate identification of three MS-H vaccine strains and nine wild-type strains. The established method thus provides a reliable and sensitive tool for discriminating between MS-H vaccine and wild-type strains, supporting improved surveillance and control in poultry farming.

## Introduction

*Mycoplasma synoviae* (MS) is an important avian pathogen associated with infectious synovitis, respiratory disorders, airsacculitis, reduced egg production, and eggshell apex abnormalities (EAA). With substantial economic implications for the global poultry industry, numerous countries have established surveillance and eradication programs targeting MS infection [1,2]. Vaccination remains a key control strategy, among which the temperature-sensitive live attenuated vaccine strain MS-H, originally developed in Australia, has been widely adopted in poultry farms and shown to be effective in managing MS outbreaks [3]. Administered via ocular or spray routes, MS-H selectively colonizes the upper respiratory tract of chickens [4]. It competitively excludes field virulent strains through niche occupation and induces local mucosal immune responses, thereby effectively suppressing the infection, replication, and transmission of wild-type strains and preventing clinical disease.

The MS-H vaccine strain was derived from the wild Australian strain 86079/7NS through chemical mutagenesis. Genetic analyses of the *vlhA* and *obg* genes indicate that most circulating strains exhibit homology with the vaccine strain [4]. A single nucleotide polymorphism (SNP) at position 367 (G/A) in the MS *obg* gene has been commonly utilized in previous studies to differentiate MS-H vaccine strains from wild-type strains [5,6]. Conventional detection of this SNP has relied on techniques such as HRM curve analysis or mismatch amplification mutation assay (MAMA) [7,8].

Recently, Pyrococcus furiosus Argonaute (PfAgo), a prokaryotic Argonaute protein derived from archaea, has attracted attention for its sequential cleavage capability [9]. PfAgo is guided by a 5′-phosphorylated DNA guide (gDNA) that is complementary to the target sequence. It cleaves the opposite strand between the 10th and 11th nucleotides relative to the guide’s 5′ end. The resulting cleavage product, carrying a new 5′-phosphate, can serve as a secondary gDNA. By coupling this with a complementary molecular beacon, PfAgo mediates a second cleavage that separates fluorophore from quencher, thereby generating a detectable fluorescent signal [10]. Owing to its high specificity in both guide activation and cleavage steps, PfAgo has been successfully applied for the detection of single-nucleotide variants [11].

Polymerase chain reaction (PCR) is a widely adopted nucleic acid amplification method, though it generally requires gel electrophoresis for result interpretation [12]. SNP detection via PCR often necessitates subsequent sequencing, increasing both time and labor. In this study, we aimed to combine PfAgo’s sequential cleavage activity and high specificity with PCR amplification, targeting the obg gene SNP at position 367. Our goal was to establish a reliable method for discriminating MS-H vaccine strains from wild-type strains. Our results demonstrate that this integrated approach exhibits good specificity and effectively distinguishes between MS-H and wild-type strains. This method may assist farms in implementing timely control measures upon MS detection, thereby blocking transmission pathways and supporting safe, efficient poultry production.

## Materials and methods

### Bacterial strains and clinical samples

The following strains were maintained in our laboratory: *Escherichia coli* (ATCC 25922), *Staphylococcus aureus* (ATCC 29213), *Klebsiella pneumoniae* (ATCC 13883), *Salmonella enterica* (ATCC 14028), the MS-H live attenuated vaccine strain, *Mycoplasma gallisepticum* S6, *Mycoplasma gallinaceum* (MGC), and *Mycoplasma gallinarum* (MGn). The *Mycoplasma synoviae* reference strain WVU1853 was kindly provided by Foshan University (Foshan, China). Throat and tracheal swab samples were collected from a poultry farm previously vaccinated with the MS-H vaccine and stored in phosphate-buffered saline (PBS).

### DNA extraction

For *Escherichia coli* (*E. coli*), *Salmonella*, *Staphylococcus aureus* (*S. aureus*), and *Klebsiella pneumoniae* (*K. pneumoniae*), genomic DNA was extracted using the HiPure Bacterial DNA Kit (Magen; Cat. No. D5131). DNA from *Mycoplasma* strains and swab samples was extracted following a previously published method with minor modifications [13]. Briefly, throat and tracheal swab samples or mycoplasma cultures were centrifuged at 14,000 × g for 10 min. The supernatant was discarded, and the pellet was resuspended in 25 µL of ddH_2_O. The suspension was heated at 100 °C for 10 min, immediately chilled on ice for 10 min, and centrifuged again at 14,000 × g for 10 min. The resulting supernatant was collected as template DNA and stored at –20 °C for subsequent analysis.

### Preparation of recombinant plasmids

Primers were designed based on the *obg* gene sequences of the MS-H vaccine strain and the MS-WVU1853T wild-type strain (S1 Table). A 1275 bp target fragment was amplified by PCR and purified using a PCR product purification kit (Vazyme, Nanjing, China). The fragment was then ligated into the pMD-19T vector (Takara, Beijing, China). The ligation product was transformed into *E. coli* DH5α competent cells (Genesand, Beijing, China). Clones exhibiting the expected PCR band size were selected and sent to Tsingke Biotech (Beijing, China) for sequencing. Plasmids from sequence-confirmed clones were extracted using the HiPure Plasmid Extraction Kit (Magen, Guangzhou, China), and their concentrations were measured with a NanoDrop spectrophotometer (NanoDrop Technologies, DE, USA). The copy numbers of the resulting plasmids, pMD-MS-H and pMD-MS-W, were calculated using the formula: copies/µL = (6.02 × 10^23^) × (concentration in ng/µL × 10^−9^)/ (DNA length × 660). Purified plasmids were stored at –20 °C until further use.

### Screening of identified substrates

**Fig 1:**
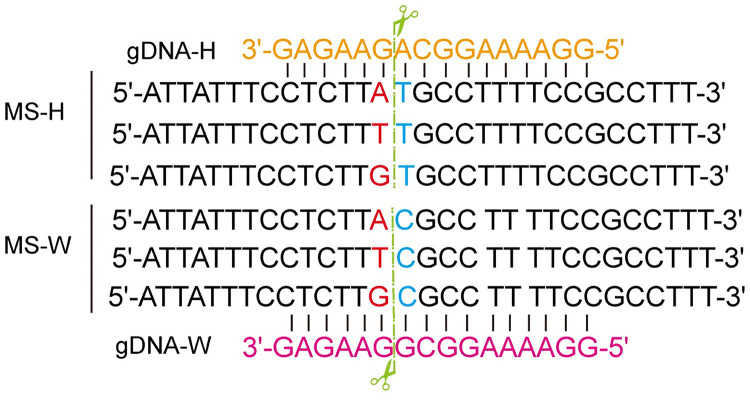
Schematic diagram of substrate screening for identification of the MS-H vaccine strain and the MS-W wild-type strain (SNP sites are indicated in blue; introduced mutant bases are shown in red).

The discrimination between the MS-H vaccine strain and the MS-W wild-type strain relies on the A367G SNP in the MS *obg* gene. Based on the stepwise cleavage mechanism of PfAgo, secondary guide DNAs (gDNA-H and gDNA-W) and corresponding substrate strands were designed for each strain. Mutations (A, T, or G) were introduced at the 10th and 11th nucleotides upstream of the SNP site within the substrate strands relative to the 5′ end of the secondary gDNAs (S1 Table). Each gDNA was used to direct PfAgo cleavage of its respective mutated substrate strand (Fig 1). The 25 µL PfAgo cleavage reaction contained: 2.5 µL 10 × buffer, 0.2 µM PfAgo, 0.6 µM gDNA, 2 µM MnCl_2_, 0.8 µM single-stranded DNA substrate, and ddH_2_O to volume. After mixing and brief centrifugation, reactions were incubated at 95 °C for 1 h. Products were combined with 2 × loading buffer and separated on a 16% urea-TBE-PAGE gel. Following electrophoresis, gels were stained in 1 × TBE buffer containing 4S GelRed nucleic acid dye for 10–15 min and visualized using a gel imaging system. Substrate strands that were cleaved specifically by gDNA-H (for MS-H) but not by gDNA-W (for MS-W), and vice versa, were subsequently labeled with fluorophore and quencher groups for downstream detection.

### PCR primer screening

Based on the selected substrate sequences, corresponding molecular probes and primary guide DNAs (gDNAs) were synthesized. Four pairs of PCR primers were designed flanking the A367G SNP in the *obg* gene (S1 Table). PCR amplification was performed using pMD‑MS‑H and pMD‑MS‑W plasmids as templates. Each 25 μL reaction contained 12.5 μL of PCR Master Mix, 1 μL each of forward and reverse primers, 2 μL of template, and ddH_2_O to volume. The thermal cycling protocol consisted of initial denaturation at 95 °C for 5 min, followed by 35 cycles of 95 °C for 30 s, 50 °C for 30 s, and 72 °C for 30 s, with a final extension at 72 °C for 10 min.

Following PCR, PfAgo cleavage reactions were assembled in 20 μL volumes containing 2 μL of 10 × buffer, 0.64 μM PfAgo, 125 nM each of obg-gDNA-1 and obg-gDNA-2, 10 μM MgSO_4_, 0.5 μM each of obg-H-TZ and obg-W-TZ, and 10 μL of the PCR product supplemented with ddH_2_O. After mixing, reactions were incubated at 95 °C for 30 min in a quantitative PCR instrument. Primer sets that effectively discriminated between MS-H and MS-W strains were selected for downstream assays.

### Validation of discriminate methods

**Sensitivity assay**: The recombinant plasmids pMD-MS-H and pMD-MS-W were serially diluted tenfold from 1 × 10^8^ to 1 × 1^00^ ng/μL and used as templates for PCR amplification, while ddH_2_O served as negative control. PCR and subsequent PfAgo cleavage reactions were performed to determine the detection sensitivity.

**Specificity assay**: Genomic DNA extracted from reference strains MS WVU1853, MS-H, *S. aureus*, *E. coli*, *Salmonella*, *K. pneumonia*, MGn, MGC, and *Mycoplasma gallisepticum* S6 was used as template for PCR and PfAgo reactions to evaluate method specificity.

**Clinical sample validation**: Throat swab samples collected from an MS-H-vaccinated flock and confirmed MS-positive by PCR were analyzed by the established PCR-PfAgo assay. Simultaneously, PCR amplicons from these samples were sent to Tsingke Biotech for sequencing to verify the reliability of the detection method.

### Statistical analysis

All data were analyzed using one-way analysis of variance (ANOVA) followed by Tukey’s multiple comparison test in GraphPad Prism 5.0. Three independent biological replicates were performed for each experiment. Data are presented as mean ± SEM. Statistical significance for pairwise comparisons between experimental groups and the control group is indicated as follows: *P < 0.05, **P < 0.01, ***P < 0.001, ****P < 0.0001.

## Results

### Mechanism of the PCR-*Pf*Ago detection method

**Fig 2:**
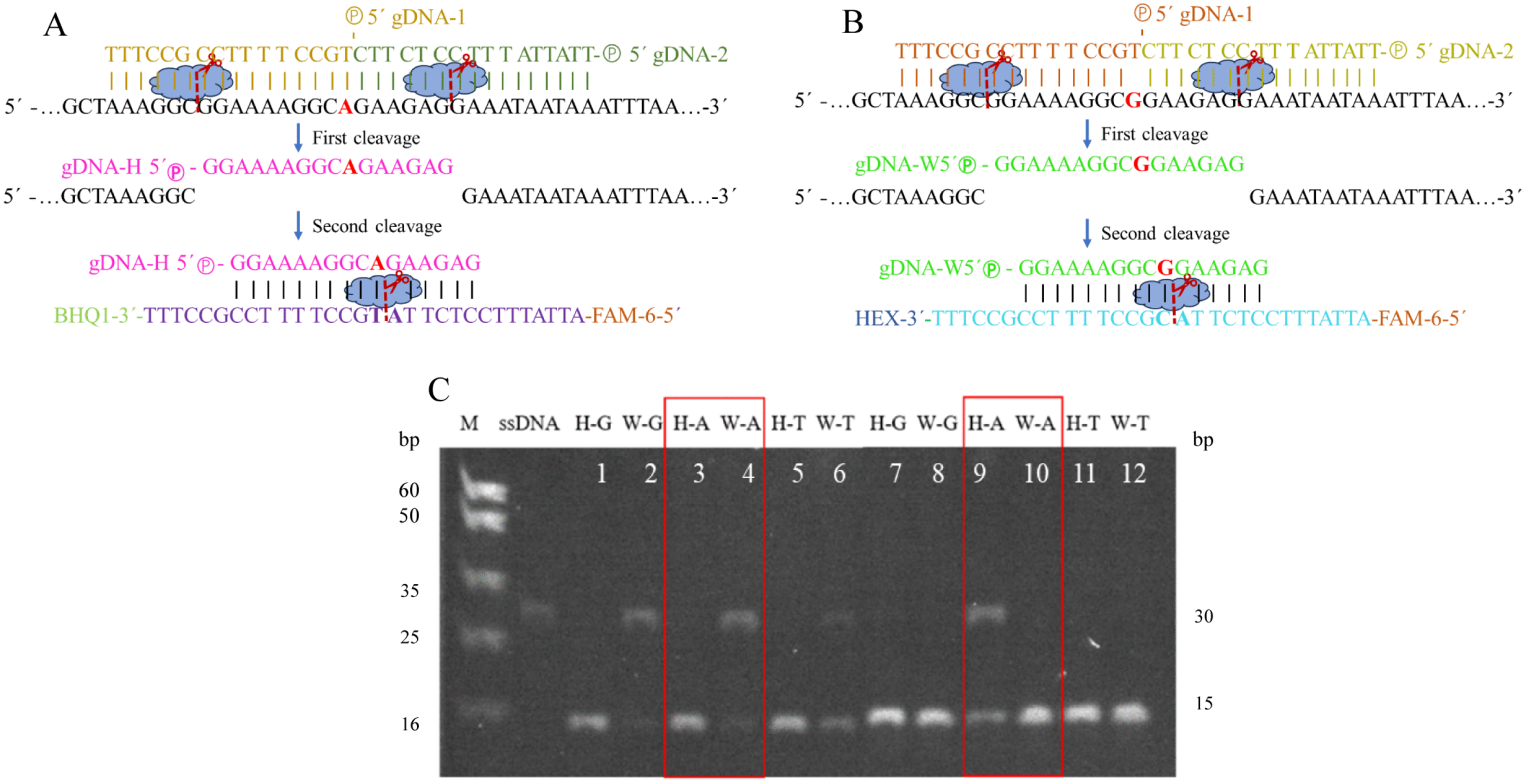
Schematic illustration of the detection process for the MS *obg* A367G SNP in (A) the MS-H vaccine strain and (B) the MS-W wild-type strain. P, phosphorylation. (C) Polyacrylamide gel electrophoresis (PAGE) analysis of substrate chain screening for MS-H and MS-W identification. Lanes 1–6: cleavage products directed by gDNA-H; lanes 7–12: cleavage products directed by gDNA-W. Labels H-G and W-G denote the MS-H and MS-W substrate chains carrying an introduced G mutation, respectively (analogous naming applies to other introduced bases). FAM, fluorophore; Q, BHQ1 quencher.

To discriminate between the MS-H vaccine strain and wild-type strains, we developed a PCR-PfAgo assay targeting the A/G single-nucleotide polymorphism (SNP) at position 367 of the *obg* gene (Fig 2A and 2B). In this system, primary guide DNAs (gDNAs) complementary to the target sequence direct PfAgo to cleave the opposite strand between the 10th and 11th nucleotides relative to the 5′ end of the target as previously reported [14]. This cleavage produces a new 5′-phosphorylated fragment, which acts as a secondary gDNA (designated gDNA-H or gDNA-W, according to the allele). The secondary gDNA then guides PfAgo to cleave corresponding fluorescently labeled substrate strands that carry allele-specific mutations and are modified with a fluorophore and a quencher.

To ensure precise SNP discrimination, the primary gDNA was designed according to the stepwise cleavage feature of PfAgo, such that the SNP site corresponds to the 10th base at the 5′ end of the secondary gDNA. Based on a previously reported strategy to enhance allelic discrimination, we then introduced A, T, or G substitutions at the adjacent 11th base in the substrate strand [14]. Two consecutive mismatches significantly impair PfAgo cleavage activity, thereby enabling allele-specific detection. Secondary gDNA-H and gDNA-W were used to cleave the corresponding mutant substrate strands derived from MS-H and MS-W templates, and the cleavage products were resolved by 16% urea-TBE-PAGE to distinguish between the two strains.

As shown in Fig 2C, when an A substitution was introduced into the substrate strand, secondary gDNA-H cleaved the MS-H-derived substrate but not the MS-W-derived substrate, while gDNA-W cleaved the MS-W substrate but not the MS-H substrate. This result demonstrates that the assay effectively discriminates between the MS-H vaccine strain and wild-type MS-W. Based on these findings, the selected substrate strands were subsequently labeled with a fluorophore and a quencher for downstream fluorescence-based detection.

### Screening of PCR primers for MS-H and MS-W identification

**Fig 3:**
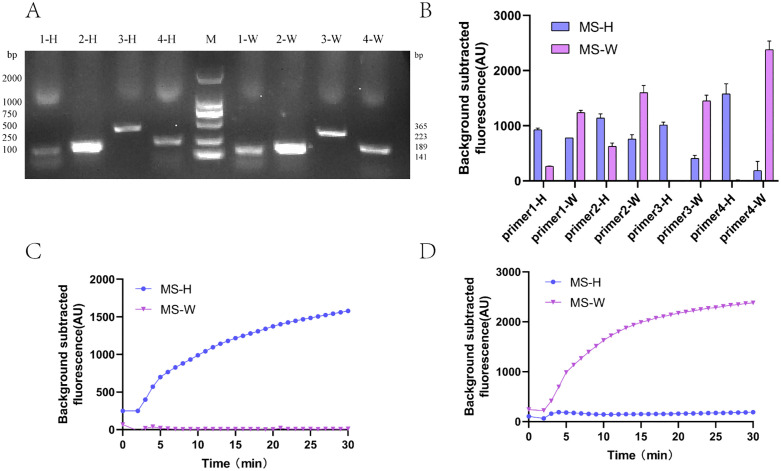
Screening of PCR primers for discrimination between MS-H and MS-W. (A) Gel electrophoresis images showing amplification of pMD-MS-H and pMD-MS-W using four different primer sets. Lane M: DNA ladder. Lanes 1–4: pMD-MS-H template amplified with primer sets 1–4, respectively. Lanes 5–8: pMD-MS-W template amplified with primer sets 1–4, respectively. The four amplicon sizes are 141 bp (primer set 1), 189 bp (primer set 2), 365 bp (primer set 3), and 223 bp (primer set 4). (B) Fluorescence signals obtained when combining each primer set with the PfAgo detection system for MS-H and MS-W. (C) Real-time fluorescence curves generated by Primer Set 4, demonstrating specific identification of MS-H and MS-W strains.

Four pairs of PCR primers were designed flanking the A367G SNP in the *obg* gene. The successfully constructed recombinant plasmids pMD‑MS‑H and pMD‑MS‑W were verified by agarose gel electrophoresis (Fig S1 in S1 Raw Images). Using these plasmids as templates, all primer sets successfully amplified the expected target bands, corresponding to 141 bp, 189 bp, 365 bp, and 223 bp, respectively (Fig 3A). Subsequent PfAgo‑based cleavage was performed to evaluate the ability of each primer pair to distinguish between the MS‑H vaccine strain and the wild‑type MS‑W strain (Fig 3B).

While primer sets 1–3 produced amplification from both MS‑H and MS‑W templates, primer set 4 showed allele‑specific amplification, it only generated a detectable signal for MS‑H when the MS‑H template was present, and only for MS‑W when the MS‑W template was used (Fig 3C). This result confirms that primer set 4 effectively discriminates between the two strains. Therefore, primer set 4 was selected for all subsequent PCR amplification steps.

### Analysis of sensitivity and specificity

**Fig 4:**
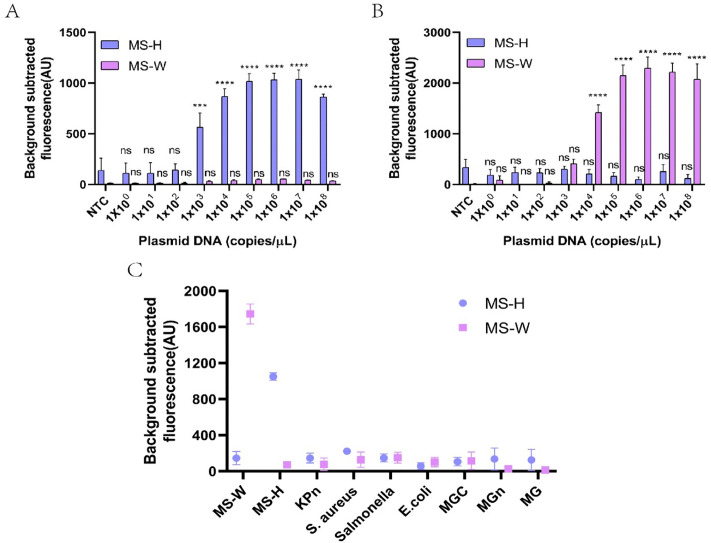
Sensitivity and specificity analysis of the PCR‑PfAgo assay for discriminating MS‑H and MS‑W strains. (A) Detection sensitivity for MS‑H. (B) Detection sensitivity for MS‑W. (C) Specificity assessment showing the fluorescence signals obtained from MS‑H, MS‑W, and other related bacterial strains.

Tenfold serial dilutions of recombinant plasmids pMD‑MS‑H and pMD‑MS‑W were prepared, ranging from 1 × 10^8^ copies/μL to 1 × 1^00^ copies/μL. Using ddH_2_O as a no‑template control, PCR amplification was performed, followed by PfAgo‑based cleavage to assess detection sensitivity. The results demonstrated that the method could detect MS‑H at a limit of 1 × 10^3^ copies/μL and MS‑W at 1 × 10^4^ copies/μL (Fig 4A and 4B).

To evaluate specificity, genomic DNA from Mycoplasma gallisepticum S6, *S. aureus*, *E. coli*, *Salmonella*, *K. pneumoniae*, MGC, MGn, MS‑WVU1853, and MS‑H were used as PCR templates. The amplicons were subsequently subjected to the PfAgo cleavage assay. As shown in Fig 4C, strong fluorescence signals were observed only for MS‑W and MS‑H, while no signal was detected for any of the non‑target strains, confirming the high specificity of the established method.

### Identification of clinical samples

**Fig 5:**
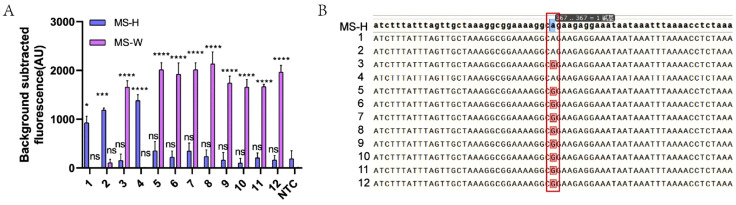
Validation of the PCR‑PfAgo assay using clinical samples for discrimination between MS‑H and MS‑W. (A) Fluorescence signals obtained from clinical samples using the PCR‑PfAgo method. (B) Sanger sequencing results of the 12 clinical samples, confirming the genotype identified by PCR‑PfAgo.

A total of 12 clinical samples previously confirmed as MS‑positive were analyzed using the established PCR‑PfAgo assay. PCR amplification was performed with ddH_2_O as a negative control, followed by PfAgo‑mediated cleavage. The results (Fig 5A) identified 3 samples as the MS‑H vaccine strain and 9 as wild‑type MS strains. To validate the assay, PCR amplicons from all samples were subjected to Sanger sequencing by Tsingke Biotechnology (Guangzhou), and the subsequent results were fully consistent with those from our established method (Fig 5B).

## Discussion

MS infection results in reduced feed conversion efficiency, diminished egg production, lower hatchability, and increased carcass condemnation rates. Chronic MS infection may also compromise the immune function of chickens, heightening susceptibility to secondary pathogens. The MS-H vaccine strain, while effective, can be transmitted horizontally within flocks and vertically through semen and the oviduct. This contributes to substantial economic losses in poultry production. By colonizing the upper respiratory tract, the MS-H strain elicits a robust immune response, significantly mitigating EAA induced by wild-type MS infection [15]. Although widely adopted in many countries for MS control, genetic analyses of *vlhA* and *obg* genes indicate that circulating strains often exhibit homology with the vaccine strain [4]. Consequently, there is a pressing need to establish sensitive molecular biology methods for monitoring MS-H vaccination and distinguishing vaccine-derived strains from wild-type infections in poultry flocks.

Currently, multiple methods have been developed to differentiate the MS-H vaccine strain from wild-type strains. These include nested PCR and high-resolution melting (HRM) curve analysis targeting a 468-nucleotide deletion in the *oppF-1* gene; dual enzyme-activated probe PCR (DEA-probes PCR), melting curve analysis, and mismatch amplification mutation assay (MAMA) based on the single-nucleotide polymorphism (SNP) at position 367 of the *obg* gene; closed-tube probe technology (CPT) relying on *vlhA* sequence polymorphisms; as well as restriction fragment length polymorphism (RFLP) combined with *vlhA* gene sequencing [15–19]. As shown in Table 1, the analytical sensitivity of our PCR-PfAgo assay (10³–10⁴ copies/µL) is lower than that of DEA-probes qPCR (~5.8 copies/µL). However, our method offers distinct advantages in terms of operational simplicity and instrument flexibility. Unlike qPCR-based approaches that require specialized real-time PCR instrumentation, our PCR-PfAgo assay can be performed using a standard thermal cycler followed by simple fluorescence detection, making it particularly suitable for resource-limited or field settings. Furthermore, the total post-PCR processing time is approximately 1.5 hours, which is substantially faster than conventional PCR followed by sequencing.

**Table 1:** Comparison of analytical sensitivity for MS-H/MS-W discrimination methods.

Method	Target Gene	Detection Limit	Reference
Our PfAgo-based assay	*obg* (SNP A367G)	MS-H: 10³ copies/µL; MS-W: 10⁴ copies/µL	This study
DEA-Probes qPCR	*obg* (SNP A367G)	5.8 copies/µL	[16]
Duplex real-time TaqMan MGB probe PCR	*ktrB* (SNP)	6.25 copies/µL	[24]
Differentiating qPCR (MS-H vs field)	*obg* (SNP A367G)	10²–10³ CFU equivalents/g	[25]
Nested PCR、HRM curve analysis	oppF-1 (468-nt deletion)	Not quantitatively reported	[15]
Melting curve analysis	*obg* (SNP A367G)	Not quantitatively reported	[17]
MAMA-PCR	*obg* (SNP A367G)	Not quantitatively reported	[18]

In recent years, Pyrococcus furiosus Argonaute (PfAgo) nucleases have garnered significant interest for their stepwise cleavage capability and high specificity, which have enabled their application in pathogen detection. However, PfAgo-based assays often require a pre-amplification step to enhance sensitivity. PCR remains a routine nucleic acid amplification technique in laboratory settings. In this study, we integrated PCR with PfAgo cleavage to shorten the overall detection time, providing results more rapidly than conventional PCR followed by sequencing. The activation of PfAgo is contingent upon guide DNA (gDNA) complementarity to its target sequence. Previous reports indicate that mismatches near the 5′ end of the gDNA do not impede PfAgo cleavage [20]. Leveraging this property, we employed the same set of primary gDNAs throughout the identification process to ensure consistent and specific detection. Our assay showed a 10‑fold sensitivity difference between alleles (MS-H: 10³ copies/µL; MS-W: 10⁴ copies/µL). This is not uncommon in PfAgo‑based SNP assays, as cleavage efficiency is sequence‑dependent (e.g., GC content, secondary structure). Similar allele‑dependent differences have been reported in other PfAgo studies, while consistent sensitivity has also been observed for different targets, confirming that such variation is sequence‑specific rather than method‑inherent [21].

The *obg* gene encodes a highly conserved GTP-binding protein, and the A367G mutation leads to the substitution of glycine by arginine at position 123 of the protein [22,23]. In this study, we use the introduced specific base mismatches into the substrate strand targeted by the secondary gDNA to enhance PfAgo’s ability to discriminate the SNP. Following optimization, an A substitution at the 11th nucleotide relative to the 5′ end of the secondary gDNA, combined with screening of PCR primers, enabled the establishment of a PfAgo-based assay that effectively distinguishes the MS-H vaccine strain from wild-type strains. Conventional identification methods typically require PCR amplification followed by sequencing, a process that often takes 1–2 days. In contrast, by exploiting the stepwise cleavage activity and high specificity of PfAgo, our integrated PCR-PfAgo method generates results within approximately 1.5 h after PCR amplification, offering a faster, more convenient, and cost-effective alternative. Specificity analysis confirmed no cross-reactivity with non-target strains, indicating high assay specificity. Furthermore, when applied to 12 MS-positive clinical samples, the results showed 100% concordance with Sanger sequencing. Nevertheless, we acknowledge several limitations that should be considered when interpreting these promising results. First, the clinical validation was performed using only 12 samples collected from a single farm. While the assay correctly discriminated all samples, this sample size is relatively small and may not fully represent the genetic and epidemiological diversity present in field populations. Second, samples containing mixed infections (both vaccine and wild-type strains) were not available for testing; therefore, the performance of the assay under co-infection scenarios remains unknown.

Despite these limitations, our findings suggest that the PCR-PfAgo assay is a promising tool for rapid MS-H and MS-W discrimination. Future studies with larger sample sets from multiple farms and geographic regions, as well as mixed infection samples, will be valuable to further validate the robustness of this method for routine clinical and field surveillance applications.

## Supporting information

S1 Table Sequences and oligonucleotides used in this study. (DOCX)

S1 Raw Images Original uncropped gel images for Fig 2C, Fig 3A and Figure S1. (DOCX)

S1 Data Raw fluorescence values for all experiments. (XLSX)

## References

[1] Kleven SH. Control of avian mycoplasma infections in commercial poultry. Avian Dis. 2008;52(3):367–74. doi: 10.1637/8323-041808-Review.1 18939621

[2] Feberwee A, de Wit S, Dijkman R. Clinical expression, epidemiology, and monitoring of Mycoplasma gallisepticum and Mycoplasma synoviae: An update. Avian Pathol. 2022;51(1):2–18. doi: 10.1080/03079457.2021.1944605 34142880

[3] Feberwee A, de Wit JJ, Landman WJM. Induction of eggshell apex abnormalities by Mycoplasma synoviae: field and experimental studies. Avian Pathol. 2009;38(1):77–85. doi: 10.1080/03079450802662772 19156584

[4] Moronato ML, Cecchinato M, Facchetti G, Mainenti M, Gobbo F, Catania S. Application of different laboratory techniques to monitor the behaviour of a Mycoplasma synoviae vaccine (MS-H) in broiler breeders. BMC Vet Res. 2018;14(1):357. doi: 10.1186/s12917-018-1669-8 30458824 PMC6245925

[5] Shen H, Wen J, Liao X, Lin Q, Zhang J, Chen K, et al. A sensitive, highly specific novel isothermal amplification method based on single-nucleotide polymorphism for the rapid detection of salmonella pullorum. Front Microbiol. 2020;11:560791. doi: 10.3389/fmicb.2020.560791 33117307 PMC7575712

[6] Wen J, Gou H, Wang S, Lin Q, Chen K, Wu Y, et al. Competitive activation cross amplification combined with smartphone-based quantification for point-of-care detection of single nucleotide polymorphism. Biosens Bioelectron. 2021;183:113200. doi: 10.1016/j.bios.2021.113200 33819904

[7] Shahid MA, Markham PF, Marenda MS, Agnew-Crumpton R, Noormohammadi AH. High-resolution melting-curve analysis of obg gene to differentiate the temperature-sensitive Mycoplasma synoviae vaccine strain MS-H from non-temperature-sensitive strains. PLoS One. 2014;9(3):e92215. doi: 10.1371/journal.pone.0092215 24643035 PMC3958494

[8] Kreizinger Z, Sulyok KM, Pásztor A, Erdélyi K, Felde O, Povazsán J, et al. Rapid, simple and cost-effective molecular method to differentiate the temperature sensitive (ts+) MS-H Vaccine Strain and Wild-Type Mycoplasma synoviae Isolates. PLoS One. 2015;10(7):e0133554. doi: 10.1371/journal.pone.0133554 26207635 PMC4514773

[9] Song J, Hegge JW, Mauk MG, Chen J, Till JE, Bhagwat N, et al. Highly specific enrichment of rare nucleic acid fractions using Thermus thermophilus argonaute with applications in cancer diagnostics. Nucleic Acids Res. 2020;48(4):e19. doi: 10.1093/nar/gkz1165 31828328 PMC7038991

[10] Xun G, Liu Q, Chong Y, Guo X, Li Z, Li Y, et al. Argonaute with stepwise endonuclease activity promotes specific and multiplex nucleic acid detection. Bioresour Bioprocess. 2021;8(1):46. doi: 10.1186/s40643-021-00401-6 38650261 PMC10991114

[11] Ye X, Zhou H, Guo X, Liu D, Li Z, Sun J, et al. Argonaute-integrated isothermal amplification for rapid, portable, multiplex detection of SARS-CoV-2 and influenza viruses. Biosens Bioelectron. 2022;207:114169. doi: 10.1016/j.bios.2022.114169 35334329 PMC9759211

[12] Bej AK, Mahbubani MH, Atlas RM. Amplification of nucleic acids by polymerase chain reaction (PCR) and other methods and their applications. Crit Rev Biochem Mol Biol. 1991;26(3–4):301–34. doi: 10.3109/10409239109114071 1718663

[13] Rasoulinezhad S, Bozorgmehrifard MH, Hosseini H, Sheikhi N, Charkhkar S. Molecular detection of Mycoplasma synoviae from backyard and commercial turkeys in some parts of Iran. Arch Razi Inst. 2018;73(2):79–85. doi: 10.22092/ari.2018.116615 30242798

[14] Su F, Zhao W, Zhao F, Cao M, Zhu T, Lv W, et al. Pyrococcus furiosus argonaute-based fluorometric biosensor for one-tube detection of cancer-associated single nucleotide polymorphisms in MicroRNAs. Anal Chem. 2025;97(8):4678–86. doi: 10.1021/acs.analchem.4c07109 39982863

[15] Zhu L, Konsak BM, Olaogun OM, Agnew-Crumptona R, Kanci A, Marenda MS, et al. Identification of a new genetic marker in Mycoplasma synoviae vaccine strain MS-H and development of a strategy using polymerase chain reaction and high-resolution melting curve analysis for differentiating MS-H from field strains. Vet Microbiol. 2017;210:49–55. doi: 10.1016/j.vetmic.2017.08.021 29103696

[16] Ogino S, Munakata Y, Ohashi S, Fukui M, Sakamoto H, Sekiya Y, et al. Genotyping of Japanese field isolates of Mycoplasma synoviae and rapid molecular differentiation from the MS-H vaccine strain. Avian Dis. 2011;55(2):187–94. doi: 10.1637/9461-071310-Reg.1 21793432

[17] Bayatzadeh MA, Pourbakhsh SA, Ashtari A, Abtin AR, Abdoshah M. Molecular typing of Iranian field isolates Mycoplasma synoviae and their differentiation from the live commercial vaccine strain MS-H using vlhA gene. Br Poult Sci. 2014;55(2):148–56. doi: 10.1080/00071668.2013.878781 24405029

[18] Bekő K, Kreizinger Z, Sulyok KM, Kovács ÁB, Grózner D, Catania S, et al. Genotyping Mycoplasma gallisepticum by multilocus sequence typing. Vet Microbiol. 2019;231:191–6. doi: 10.1016/j.vetmic.2019.03.016 30955809

[19] Liu R, Lin Q, Cai Q, Liang Y, Xu X, Chen Q, et al. A novel high sensitive, specificity duplex enzyme-activated differentiating probes PCR method for the SNP detection and differentiation of MS-H vaccine strains from wild-type Mycoplasma synoviae strains. Poult Sci. 2024;103(8):103874. doi: 10.1016/j.psj.2024.103874 38833744 PMC11190711

[20] Liu Q, Guo X, Xun G, Li Z, Chong Y, Yang L, et al. Argonaute integrated single-tube PCR system enables supersensitive detection of rare mutations. Nucleic Acids Res. 2021;49(13):e75. doi: 10.1093/nar/gkab274 33905513 PMC8287959

[21] Zhang Y, Huo C, Zhang T, Liu Q, He P, Du J, et al. A novel Pyrococcus furiosus argonaute-based method for rapid and sensitive detection of Mycoplasma pneumoniae and a macrolide-resistance-related mutation. J Clin Microbiol. 2026;64(1):e0108925. doi: 10.1128/jcm.01089-25 41410535 PMC12802221

[22] Verstraeten N, Fauvart M, Versées W, Michiels J. The universally conserved prokaryotic GTPases. Microbiol Mol Biol Rev. 2011;75(3):507–42. doi: 10.1128/MMBR.00009-11 21885683 PMC3165542

[23] Shahid MA, Markham PF, Markham JF, Marenda MS, Noormohammadi AH. Mutations in GTP binding protein Obg of Mycoplasma synoviae vaccine strain MS-H: Implications in temperature-sensitivity phenotype. PLoS One. 2013;8(9):e73954. doi: 10.1371/journal.pone.0073954 24069254 PMC3775756

[24] Zhao L, Tang X, Guo W, Zhang B, Peng H, Ye L, et al. Using a novel gene site to develop a duplex real-time TaqMan MGB probe PCR method for the SNP detection and differentiation between the MS-H live vaccine strain and wild-type Mycoplasma synoviae strains. Poult Sci. 2025;104(5):105011. doi: 10.1016/j.psj.2025.105011 40080948 PMC11953997

[25] Dijkman R, Feberwee A, Landman WJM. Development, validation and field evaluation of a quantitative real-time PCR able to differentiate between field Mycoplasma synoviae and the MS-H-live vaccine strain. Avian Pathol. 2017;46(4):403–15. doi: 10.1080/03079457.2017.1296105 28277780

