# Supplementary material for: Pyrococcus furiosus Argonaute coupled PCR assay for accurate discrimination between the MS-H vaccine strain and clinical isolates of Mycoplasma synoviae
Source: PLoS One. 2026 Jul 28;21(7):e0351464. doi: 10.1371/journal.pone.0351464 (PMC13411903; doi:10.1371/journal.pone.0351464)
Supplement: S1 Table — (DOCX) [file pone.0351464.s001.docx]

**Pyrococcus furiosus Argonaute coupled PCR assay for accurate discrimination between the MS-H vaccine strain and clinical isolates of Mycoplasma synoviae**

Yanli Zhao^1^, Yinling Wang^1^, Ge Song^1^, Houqiang Luo^1^, Liyan Dong^1^, Qingsong Han^1^, Mengling Yang^1^, Jing Pan^1^, Hongxia Jiang^2,3^*

^1^College of Animal Science, Wenzhou Vocational College of Science and Technology, Wenzhou 325006, China.

^2^Guangdong Key Laboratory for Veterinary Pharmaceutics Development and Safety evaluation, College of Veterinary Medicine, South China Agricultural University, Guangzhou, 510642, China;

^3^Guangdong Laboratory for Lingnan Modern Agriculture, Guangzhou, China, South China Agricultural University, Guangzhou, 510642, China

* For correspondence: Hong-Xia Jiang, Ph.D.

Guangdong Key Laboratory for Veterinary Pharmaceutics Development and Safety evaluation, College of Veterinary Medicine, South China Agricultural University, Guangzhou, 510642, China.

Tel.: +86-020-85284896; Email address: hxjiang@scau.edu.cn

**Table S1.** Sequences that were used in this study

| Name | Sequence (5′-3′) | Fragment length (bp) |
| --- | --- | --- |
| MS-obg-F | ATGGCAAAGTTTATAGATCAAGT | 1275 |
| MS-obg-R | TTATAATTCTTCCTCCCATTCAA |  |
| gDNA-H | GGAAAAGGCAGAAGAG |  |
| gDNA-W | GGAAAAGGCGGAAGAG |  |
| obg-H-A | ATTATTTCCTCTT**A**TGCCTTTTCCGCCTTT |  |
| obg-H-T | ATTATTTCCTCTT**T**TGCCTTTTCCGCCTTT |  |
| obg-H-G | ATTATTTCCTCTT**G**TGCCTTTTCCGCCTTT |  |
| obg-W-T | ATTATTTCCTCTT**T**CGCCTTTTCCGCCTTT |  |
| obg-W-A | ATTATTTCCTCTT**A**CGCCTTTTCCGCCTTT |  |
| obg-W-G | ATTATTTCCTCTT**G**CGCCTTTTCCGCCTTT |  |
| obg-PCR-F1 | AATCATCTTTATTTAGTTGCT | 141 |
| obg-PCR-R1 | AATTTTTAATACGATATTAGC |  |
| obg-PCR-F2 | GTTAAAGTTCCGCTTGGAACGCTGG | 189 |
| obg-PCR-R2 | AGCTTCGTATTTTTCTCCGGGCATT |  |
| obg-PCR-F3 | AAGGAGGTGATGGGATGATTTC | 365 |
| obg-PCR-R3 | AGGAGCGGTGTTTTTAGAGGTT |  |
| obg-PCR-F4 | GCTAAAGGAAAAGATACCAT | 223 |
| obg-PCR-R4 | TTAATACGATATTAGCTTCG |  |
| obg-gDNA-1 | TGCCTTTTCCGCCTTT |  |
| obg-gDNA-2 | TTATTATTTCCTCTTC |  |
| obg-H-TZ | FAM-attatttcctcttatgccttttccgccttt-BHQ1 |  |
| obg-W-TZ | HEX-attatttcctcttacgccttttccgccttt-BHQ2 |  |

Note: Bases in bold are introduced mutations; bases highlighted in red denote the SNP locus.
