# Supplementary material for: Pyrococcus furiosus Argonaute coupled PCR assay for accurate discrimination between the MS-H vaccine strain and clinical isolates of Mycoplasma synoviae
Source: PLoS One. 2026 Jul 28;21(7):e0351464. doi: 10.1371/journal.pone.0351464 (PMC13411903; doi:10.1371/journal.pone.0351464)
Supplement: S1 Raw Images — (DOCX) [file pone.0351464.s002.docx]

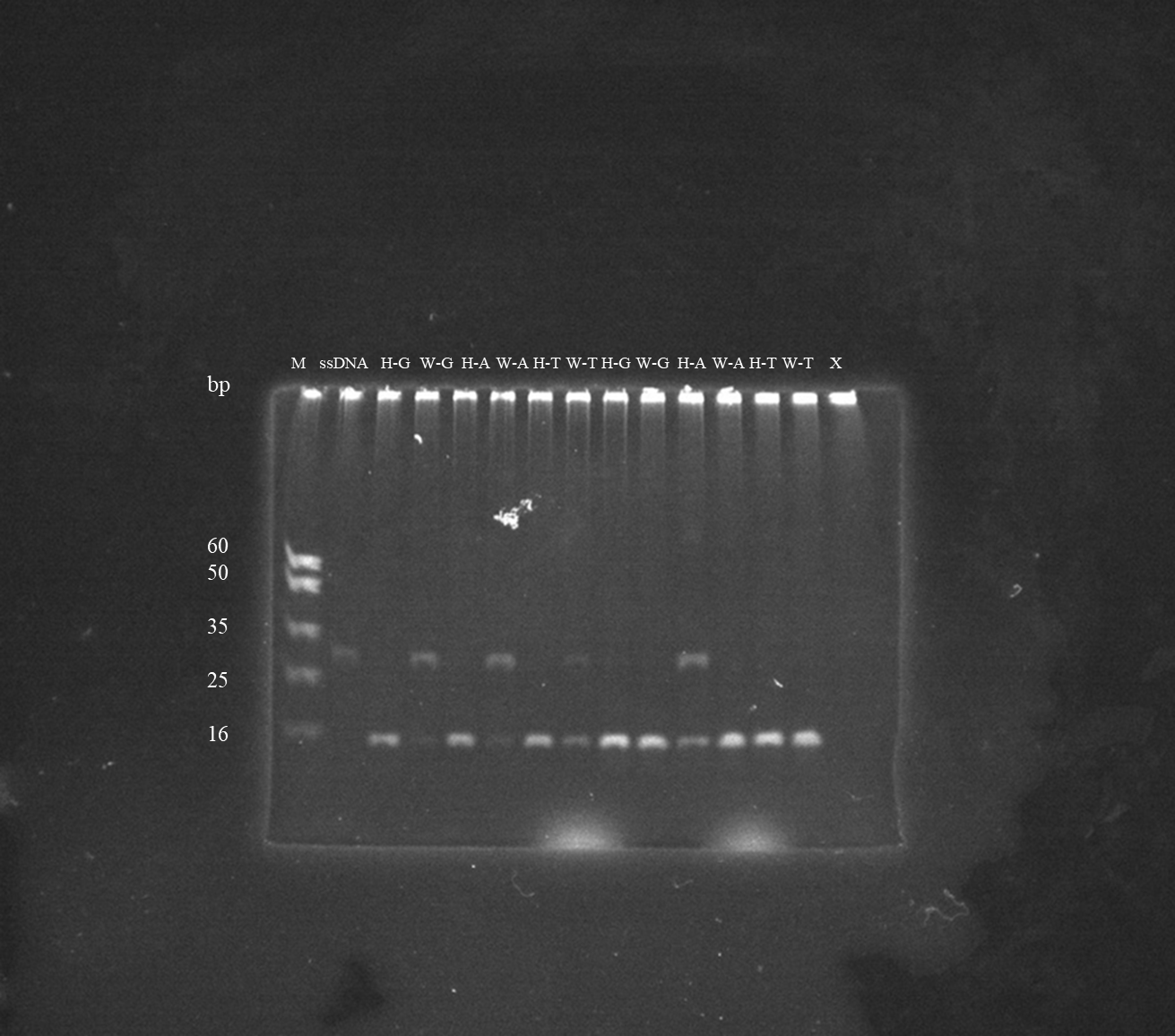


**Fig 2C. Screening of substrate chains for MS-H and MS-W identification.** Polyacrylamide gel electrophoresis (PAGE) analysis of substrate chain screening. M: DNA marker.


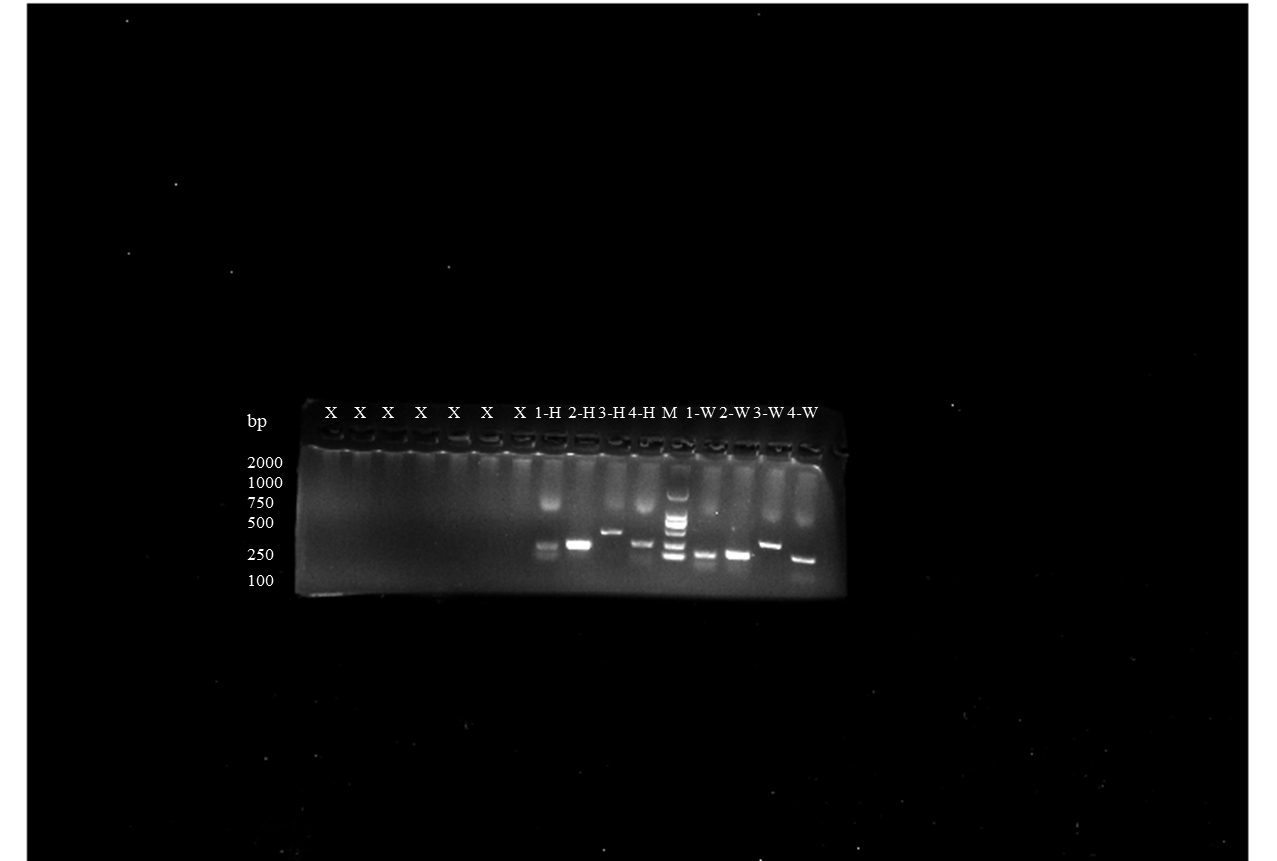


**Figure 3A.** **Screening of PCR primers.** Gel electrophoresis images showing amplification of pMD-MS-H and pMD-MS-W using four different primer sets. M: DNA marker.


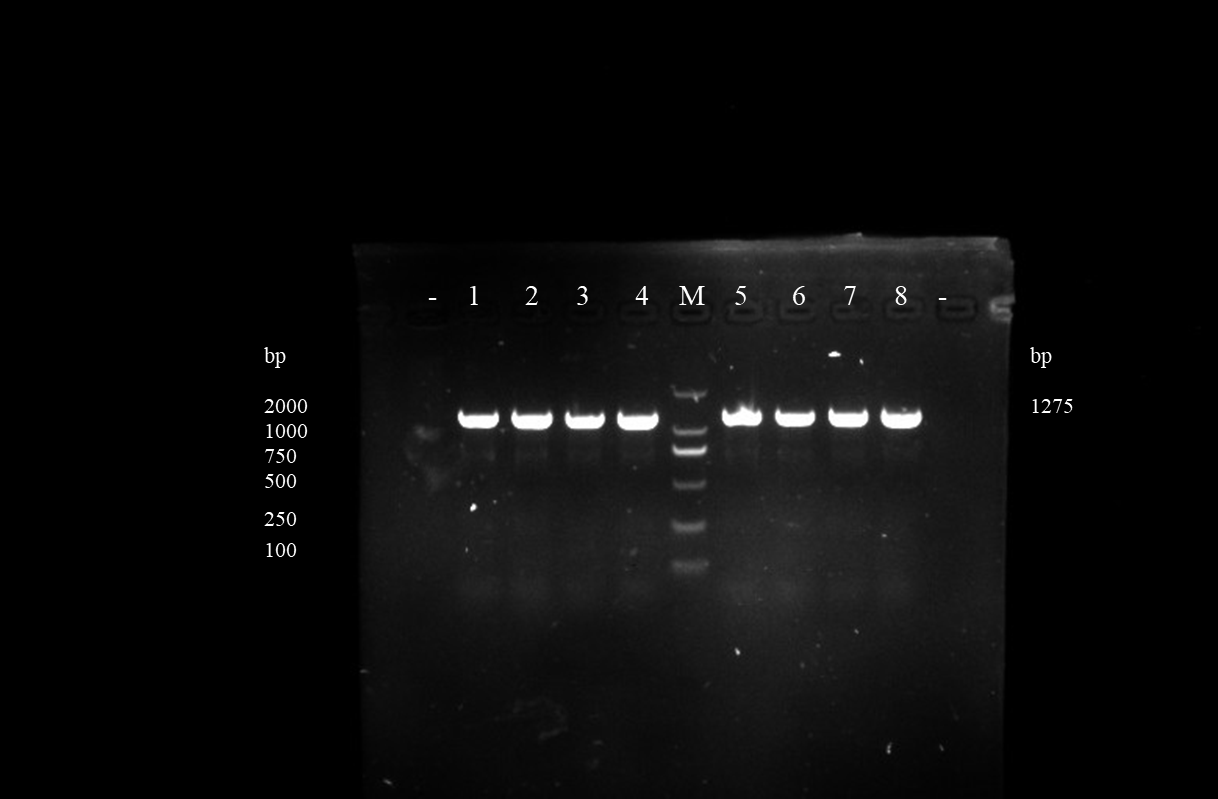


**Figure S1.** **Identification of recombinant plasmids by agarose gel electrophoresis.** Lanes 1-4: pMD-MS-H recombinant plasmid; lanes 5-8: pMD-MS-W recombinant plasmid; -: negative control. M: DNA marker.
